# Intermittent hypoxia conditioning as a potential prevention and treatment strategy for ischemic stroke: Current evidence and future directions

**DOI:** 10.3389/fnins.2022.1067411

**Published:** 2022-11-25

**Authors:** Honghua Yuan, Jia Liu, Yuhang Gu, Xunming Ji, Guangxian Nan

**Affiliations:** ^1^Department of Neurology, China-Japan Union Hospital of Jilin University, Changchun, China; ^2^Beijing Institute of Brain Disorders, Laboratory of Brain Disorders, Ministry of Science and Technology, Collaborative Innovation Center for Brain Disorders, Beijing Advanced Innovation Center for Big Data-based Precision Medicine, Capital Medical University, Beijing, China; ^3^Department of Neurosurgery, Xuanwu Hospital, Capital Medical University, Beijing, China

**Keywords:** intermittent hypoxia conditioning, ischemic stroke, vascular risk factors, neurovascular protection, neurovascular restoration, cerebral collaterals, circadian rhythm

## Abstract

Ischemic stroke (IS) is the leading cause of disability and death worldwide. Owing to the aging population and unhealthy lifestyles, the incidence of cerebrovascular disease is high. Vascular risk factors include hypertension, diabetes, dyslipidemia, and obesity. Therefore, in addition to timely and effective reperfusion therapy for IS, it is crucial to actively control these risk factors to reduce the incidence and recurrence rates of IS. Evidence from human and animal studies suggests that moderate intermittent hypoxia (IH) exposure is a promising therapeutic strategy to ameliorate common vascular risk factors and comorbidities. Given the complex pathophysiological mechanisms underlying IS, effective treatment must focus on reducing injury in the acute phase and promoting repair in the recovery phase. Therefore, this review discusses the preclinical perspectives on IH conditioning as a potential treatment for neurovascular injury and highlights IH pre and postconditioning strategies for IS. Hypoxia conditioning reduces brain injury by increasing resistance to acute ischemic and hypoxic stress, exerting neuroprotective effects, and promoting post-injury repair and regeneration. However, whether IH produces beneficial effects depends not only on the hypoxic regimen but also on inter-subject differences. Therefore, we discuss the factors that may influence the effectiveness of IH treatment, including age, sex, comorbidities, and circadian rhythm, which can be used to help identify the optimal intervention population and treatment protocols for more accurate, individualized clinical translation. In conclusion, IH conditioning as a non-invasive, non-pharmacological, systemic, and multi-targeted intervention can not only reduce brain damage after stroke but can also be applied to the prevention and functional recovery of IS, providing brain protection at different stages of the disease. It represents a promising therapeutic strategy. For patients with IS and high-risk groups, IH conditioning is expected to develop as an adjunctive clinical treatment option to reduce the incidence, recurrence, disability, and mortality of IS and to reduce disease burden.

## Introduction

With an aging population and unhealthy lifestyles, cerebrovascular disease morbidity and mortality rates are gradually increasing, and the disease burden is increasing (Tsao et al., 2022). Ischemic stroke (IS) is the most common cerebrovascular disease and the main cause of death and disability. At present, venous thrombolysis and mechanical thrombectomy are the only two effective methods for the treatment of acute IS (Xiong et al., 2022). Although many neuroprotective drugs have been tested in preclinical trials with animal models to reduce stroke injury, they have not been successfully transferred to the clinical setting (Lyden, 2021). Therefore, it is urgent to identify alternative treatment methods to improve the brain’s self-protection capacity. It is also critical to actively control the risk factors for cerebrovascular disease, to reduce its incidence and recurrence rate.

Over the last two decades, the benefits of adaptive regulation have been widely reported (Gidday, 2006; Sommer, 2009). Preconditioning refers to the use of short-term sub-lethal stimuli to improve the tolerance of cells, tissues, and organs to subsequent lethal damage, thus playing an endogenous protective role (Dirnagl et al., 2009). Many preclinical studies have shown that preconditioning schemes, such as hypoxia, ischemia, hypothermia, drugs, and exercise can induce adaptive endogenous protective pathways and exert protective effects against central nervous system diseases, but the exact mechanism underlying these effects remains unclear (Obrenovitch, 2008; Stetler et al., 2014; Stevens et al., 2014; Li et al., 2017; Hafez et al., 2021). Hypoxia conditioning, as a simple, non-invasive, systemic intervention treatment method that does not involve the use of drugs, has more extensive application value than other preconditioning schemes and has great therapeutic potential for a variety of clinical diseases (Navarrete-Opazo and Mitchell, 2014; Mateika et al., 2015; Verges et al., 2015).

Moderate and well-controlled mild intermittent hypoxia (IH) refers to a short period of daily alternating exposure to normobaric hypoxia and normoxia (or hyperoxia) for several weeks (Burtscher et al., 2022). Moderate IH triggers adaptive phenomena that produce beneficial therapeutic effects, improving the body’s defense ability against future potential damage in the future (Arkhipenko et al., 2005; Manukhina et al., 2013). Moreover, this type of repetitive moderate IH provides better safety and lasting treatment outcomes compared to acute or persistent hypoxia modes (Zhu et al., 2007; Stowe et al., 2011; Terraneo et al., 2017).

Increasingly, experimental and clinical studies indicate that IH can not only reduce the severity of injury after IS (Stowe et al., 2011; Monson et al., 2014; Selvaraj et al., 2017), but also decrease the risk factors for cerebrovascular disease (Lyamina et al., 2011; Urdampilleta et al., 2012; Serebrovska et al., 2017), suggesting that IH intervention can increase the body’s ability to resist injury and promote repair, reduce the risk of developing IS, and exert neurological and cerebrovascular protective effects. Therefore, this paper summarizes the relevant supporting research evidence and proposes the feasibility that IH can be applied to the prevention, treatment, and rehabilitation of IS. This treatment method has great translational prospects, but more basic and clinical studies are still needed to elucidate its mechanism of brain protection and other potentially beneficial effects. Further, the optimal hypoxia therapy protocol requires investigation for safe application to patients and high-risk populations in a clinical settings.

In clinical translational studies of IH, it is noteworthy that IS occurs more often in older adults, more often in males than in females, and most of the affected individuals have comorbidities (Branyan and Sohrabji, 2020; Candelario-Jalil and Paul, 2021). Therefore, it should be noted that the therapeutic effects of IH may vary among individuals. In addition, the opposite circadian rhythms of humans and rodents, which are normally used in experimental studies, may also affect the relative effectiveness of the treatment (Esposito et al., 2020; Lo et al., 2021). However, it is unclear whether the above-mentioned factors affect the therapeutic effect of IH. Taking these factors into account, future experimental and clinical studies can provide more comprehensive and robust evidence for individualized IH treatment. It is hoped that the hypoxia intervention program will be continuously optimized in the future to reduce the incidence of cerebrovascular disease, decrease the disability and mortality rates associated with IS, improve the functional prognosis of stroke patients, and enhance quality of life.

## Intermittent hypoxia: A therapeutic hypoxia protocol

Intermittent hypoxia is a very promising strategy for treating and preventing human diseases that has attracted increasing attention in various fields. Many clinical studies have shown that intermittent exposure to moderate hypoxia can have beneficial effects on both sick and healthy individuals (Zhang et al., 2010; Leone and Lalande, 2017; Liu et al., 2017; Wojan et al., 2021). IH is also safe and effective for the older adults, for whom short periods of alternating exposure to moderate hypoxia and normoxia environments can alter body composition and health status by improving exercise tolerance, metabolism, inflammation, and systemic arterial pressure (Burtscher et al., 2004; Timon et al., 2021, 2022). Studies focusing on central nervous system diseases have shown that hypoxia conditioning can promote health in the aging brain (Burtscher et al., 2021a) and make it more resistant to acute brain injury (Miller et al., 2001; Lin et al., 2003; Stowe et al., 2011, 2012; Wacker et al., 2012a,b), as well as play a protective role against chronic age-related neurodegenerative diseases, such as cognitive impairment (Bayer et al., 2017; Serebrovska et al., 2019c; Wang et al., 2020), Alzheimer’s disease (Manukhina et al., 2016), and Parkinson’s disease (Burtscher et al., 2021b). IH is expected to become a new therapeutic strategy against aging and neurodegeneration. However, it should be noted that the beneficial effects of hypoxia on the body and its effects as disease treatment depend on factors, such as the concentration, mode, and exposure time of hypoxia.

### Hypoxia is a double-edged sword: “dose” is the key

The metabolism of the brain is highly active. Oxygen consumption of the brain accounts for 20% of the amount consumed by the whole body in the resting state. It is very sensitive to changes in oxygen concentration (Magistretti and Allaman, 2015). Therefore, the brain is especially vulnerable to the adverse effects of hypoxia. When an organism first enters into a high-altitude area, acute exposure to low oxygen partial pressure is likely to cause various symptoms collectively known as acute altitude illnesses, among which high-altitude brain edema can be life-threatening (Luks and Hackett, 2022). High-altitude hypoxia environments exert certain effects on the central nervous system of individuals habituated to low altitudes (Wilson et al., 2009), which may lead to cognitive dysfunction (Wang et al., 2022), sleep disorders, and may also induce and aggravate central nervous system diseases (Falla et al., 2021). Hypoxia has also been increasingly recognized as an important factor in the development of neurodegenerative diseases (Burtscher et al., 2021a). During IS, oxygen delivery to the brain is impaired due to vascular occlusion, resulting in neurological deficits. The above shows that maladaptive responses to hypoxia and local severe hypoxia can cause damage to important organs, which is harmful to human health.

In contrast, moderate hypoxia adaptation is beneficial to the body. “Altitude training” and training combined with normobaric hypoxia can enhance physical fitness through hypoxia stimulation, improving hypoxia endurance and sports performance (Flaherty et al., 2016). An epidemiological study found low mortality rates from stroke and coronary heart disease in high-altitude areas of Switzerland (Faeh et al., 2009). High-altitude rodents obtain abundant collateral circulation through gene selection, which can prevent tissue damage after brain, coronary artery, and peripheral artery occlusion (Faber et al., 2021). Many experimental studies in rodents have shown that hypoxic preconditioning can protect important organs (including the heart and brain) from fatal cell damage caused by hypoxia or ischemia (Stowe et al., 2011; Manukhina et al., 2013). Thus, non-lethal moderate hypoxic stimulation as a means of enhancing hypoxic adaptation, which subsequently protects important organs and tissues from similar but more severe damage, is a promising therapeutic strategy.

In summary, hypoxia can induce either physiological adaptation or pathological injury, including death. This is because when oxygen delivery is interrupted or reduced, body cells sense the reduced oxygen supply and respond by initiating endogenous protection through adaptive mechanisms to promote cell survival under hypoxic conditions. However, when the intensity of hypoxia exceeds their maximum tolerance, a cascade of gene expression cascades (like a chain reaction) is initiated, subsequently leading to altered cellular function or even death (Terraneo et al., 2017; Lee et al., 2020; Burtscher et al., 2022). Therefore, whether hypoxia is beneficial or harmful to the body largely depends on the “dose” of hypoxia (Jackman et al., 2014; Navarrete-Opazo and Mitchell, 2014; Serebrovska et al., 2016). Low-dose intermittent exposure to hypoxia (FiO_2_ = 9–16%), 3–15 times per day, has been proven to be beneficial in clinical and animal experiments (Navarrete-Opazo and Mitchell, 2014). In addition, it is important to note that differences in hypoxia tolerance between individuals can also affect the outcome of hypoxia (Dempsey and Morgan, 2015).

### Different forms of intermittent hypoxia conditioning

**FIGURE 1:**
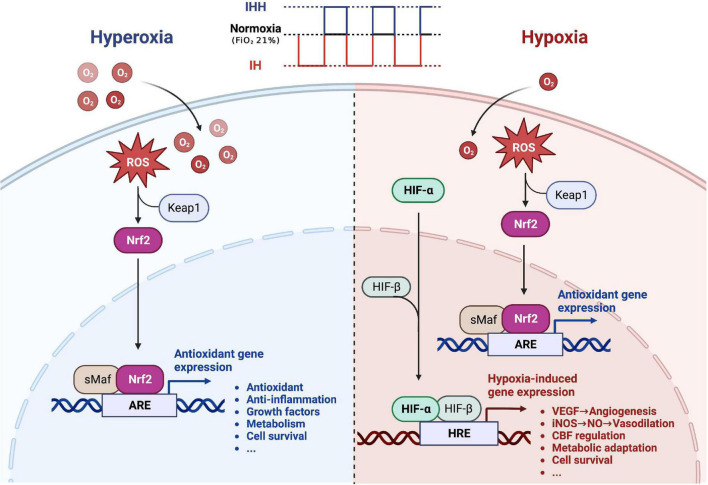
The potential therapeutic benefit of intermittent hypoxia conditioning or intermittent hypoxia-hyperoxia conditioning for cerebrovascular protection. During intermittent hypoxia (IH) conditioning or intermittent hypoxia-hyperoxia (IHH) conditioning, moderate amounts of ROS are produced, activating the transcription factor Nrf2, which promotes the synthesis of protective proteins and improves the brain’s defense response to severe ischemic-hypoxic injury. In addition, hypoxia activates the transcription factor HIF, which induces numerous genes essential for cell metabolism, proliferation, and survival. Many of these genes play a central role in injury tolerance and promotion of tissue oxygenation, such as vascular endothelial growth factor (VEGF), and inducible NO synthase (iNOS). Replacing normoxia with hyperoxia during the reoxygenation phase can amplify the beneficial effects of intermittent hypoxia. ROS, reactive oxygen species; Nrf2, nuclear factor erythroid 2-related factor 2; ARE, antioxidant response element; HIF, hypoxia-inducible factor; HRE, hypoxia-responsive element. Created with www.biorender.com.

Therapeutic IH refers to short-term alternating exposure to normobaric hypoxia and normoxia (or hyperoxia) (Navarrete-Opazo and Mitchell, 2014; Mateika et al., 2015; Serebrovska et al., 2016). As a moderate and non-harmful stressor, it can promote adaptive responses and exert various beneficial effects on physical health (Gangwar et al., 2020; Behrendt et al., 2022), avoiding the possible harmful effects of continuous hypoxia. Additionally, IH can also improve the defense against potential future damage by initiating endogenous protective mechanisms (Stowe et al., 2011; Manukhina et al., 2013, 2016, 2018; Mallet et al., 2018; Burtscher et al., 2021a; Su et al., 2022). Intermittent hypoxia-hyperoxia (IHH) is a modified IH protocol. The normoxia period is replaced by a moderate hyperoxia period (FiO_2_ = 30–40%), resulting in a faster recovery from deoxygenation (Bayer et al., 2017; Glazachev et al., 2017; Dudnik et al., 2018; Serebrovska et al., 2019c,^2022^; Afina et al., 2021; Behrendt et al., 2022; Bestavashvili et al., 2022). Compared with IH, IHH offers a more obvious improvement in the clinical parameters and is considered to offer more beneficial effects (Serebrovska et al., 2019a). The underlying mechanism may be related to a more pronounced induction of reactive oxygen species during mild hyperoxia, triggering an intracellular redox signaling cascade that induces the synthesis of intracellular protective proteins with antioxidant and anti-inflammatory effects through the activation of the transcription factors nuclear factor erythroid 2-related factor 2 (Nrf2) and hypoxia-inducible factor (HIF) (Arkhipenko et al., 2005; Serebrovska et al., 2017; Burtscher et al., 2022). Based on the limited animal and clinical studies available, we summarized the potential therapeutic benefits of IH conditioning or IHH conditioning on cerebrovascular protection in Figure 1. The protective mechanism of IH and IHH is not completely understood yet, and further research on their molecular details is needed. In addition, IH therapy includes IH exposure and IH training (IHT). The latter consists of a combination of exercise and IH exposure (Morishima et al., 2015; Timon et al., 2021), while the former has a wider range of applications and is more appropriate for groups that, for various reasons, are not suitable for exercise.

## Intermittent hypoxia conditioning improves common risk factors of ischemic stroke

Ischemic stroke is often accompanied by hypertension, diabetes, dyslipidemia, and obesity; and these comorbidities further complicate the pathology of IS (Gottesman and Seshadri, 2022). These patients often need a combination of oral medications to control their risk factors, which may bring unavoidable side effects. Therefore, in addition to modifying an unhealthy lifestyle, it is very important to identify effective non-pharmacological interventions for cardiovascular and cerebrovascular diseases and for metabolic risk factors that can be applied as adjunctive therapies for people at high risk for cerebrovascular disease. A growing number of clinical and experimental findings suggest that IH can modulate risk factors associated with cerebrovascular disease (Serebrovskaya et al., 2008; Urdampilleta et al., 2012; Serebrovska et al., 2017, 2019a; Kayser and Verges, 2021), which is a potential therapeutic strategy. This evidence supports the applicability of IH in the preventive treatment of IS.

### Intermittent hypoxia and hypertension

Inadequate production and reduced availability of nitric oxide lead to increased blood pressure (Panza et al., 1993), and long-term chronic hypertension is a common risk factor for stroke. Experimental studies on rodents show that the hypotensive effect of IH in spontaneously hypertensive rats is due to the stimulation of nitric oxide synthesis and storage in blood vessels (Manukhina et al., 2011). In addition, intermittent hypobaric hypoxia can reduce blood pressure in these rats, potentially through the inhibition of the renin-angiotensin system (Chen et al., 2021). In a renal vascular hypertensive rat model, intermittent hypobaric hypoxia also has an anti-hypertensive effect (Li et al., 2019). In human studies, intermittent normobaric hypoxic adaptation for 20 days was reported to reduce blood pressure levels in patients with stage 1 hypertension, accompanied by an increase in nitric oxide synthesis (Lyamina et al., 2011). Another study further confirmed the hypotensive effect of IH conditioning in patients with stage 1 hypertension, confirmed that hypoxic adaptation combined with exercise produced a better and longer-lasting hypotensive effect than oral antihypertensive drugs alone, and found that the hypotensive effect of hypoxia correlated with elevated levels of nitric oxide (NO) and hypoxia-inducible factor-1alpha (HIF-1α) (Muangritdech et al., 2020). Moreover, IH conditioning also provides good anti-hypertensive effects in the presence of comorbid conditions. IHH training in patients with coronary artery disease not only reduced blood pressure, but also improved exercise capacity, reduced angina attacks, enhanced the left ventricular ejection fraction, and reduced blood glucose (Glazachev et al., 2017). In patients with metabolic syndrome, IHH exposure can significantly reduce systolic and diastolic blood pressure (Bestavashvili et al., 2022). Mild IH can reduce blood pressure in patients with obstructive sleep apnea (OSA) complicated with hypertension (Panza et al., 2022). Evidence from studies in animals and humans (Tables 1, 2) suggests that well-controlled mild IH conditioning regimens may be a safe and effective way to prevent and treat hypertension.

**TABLE 1:** Effects of intermittent hypoxia on vascular risk factors in humans.

References	Participants	Hypoxia protocols	Results
(Bestavashvili et al., 2022)	65 patients with MS: IHH group *n* = 32 (age 56.9 ± 11.7), control group *n* = 33 (age 59.8 ± 10.3)	IHH: 5–8 cycles of 4–7 min hypoxia (11–12%) followed by 2–4 min hyperoxia (30–35%), 5 sessions/week for 3 weeks	SBP↓, DBP↓, improve lipid profile and liver functional state
(Panza et al., 2022)	16 males with OSA and HTN: IH *n* = 10 (age 40.7 ± 9.8), control *n* = 6 (age 46.2 ± 10.3)	IH: 12 cycles of 2 min hypoxia (8%) followed by 2 min normoxia, 5 sessions/week for 3 weeks	SBP↓, DBP↓, accompanied by parasympathetic↑, sympathetic↓
(Muangritdech et al., 2020)	47 HTN patients: IHR *n* = 15, IHT *n* = 15, control *n* = 17	IH: eight cycles of 3 min hypoxia (14%) followed by 3 min normoxia, 2 sessions/week for 6 weeks	SBP↓, Nox and HIF-1α were negatively correlated with SBP
(Glazachev et al., 2017)	46 CAD patients: IHH *n* = 27 (age 52–77); control = 19 (age 43–83)	IHH: 5–7cycles of 4–6 min hypoxia (10–12%) with 3 min hyperoxia (30–35%), 3 sessions/week for 8 weeks	SBP↓, DBP↓
(Lyamina et al., 2011)	37 stage 1 HTN (age ∼32)	IH: 4–10 cycles of 3 min hypoxia (10%) with 3 min normoxia, 1 session/day, for 20 days	SBP↓, DBP↓, normalization of NO production
(Afina et al., 2021)	65 patients with MS: IHH group *n* = 32 (age 44.5–65.5), control group *n* = 33 (age 56.2–66.0)	IHH: 5–8 cycles of 4–7 min hypoxia (11–12%) followed by 2–4 min hyperoxia (30–35%), 5 sessions/week for 3 weeks	Improved lipid profile and anti-inflammatory status
(Gangwar et al., 2020)	40 male healthy volunteers (age 22–25)	On day 0: 1 h hypoxia (13.5%); Days 1–4: hypoxia (12%), 4 h/day for 4 days; on day 5: 1 h hypoxia (13.5%)	Regulate lipid metabolism
(Serebrovska et al., 2019b)	11 prediabetic patients (age 48–70) and seven healthy volunteers (age 44–68)	IH: four cycles of 5 min hypoxia (12%) followed by 5 min normoxia, 3 sessions/week for 3 weeks	Normalizing blood insulin level, correlated with an enhanced mRNA expression of PDK-1 in leukocytes
(Serebrovska et al., 2019a)	55 patients with prediabetes (age 51–74): IHH group *n* = 17, IH group *n* = 22, control *n* = 16	IHH or IH: four cycles of 5 min hypoxia (12%) followed by 3 min hyperoxia (33%) or normoxia, 5 sessions/week for 3 weeks	Reduced blood glucose (fasting and OGTT); decreased total blood cholesterol and LDL
(Costalat et al., 2018)	Six overweight and obese individuals (age 56.2 ± 10)	IH: 70 min of repeated cycles of hypoxia (SpO_2_ = 70%) followed by re-oxygenation (SpO_2_ = 95%), 5 sessions/week for 2 weeks	A single IH: reduced blood glucose and lactate; 10 sessions IH: decreased LDLc, LDLc/HDLc ratio and SBP
(Serebrovska et al., 2017)	Seven healthy and 11 prediabetic individuals (age 44–70)	IH: four cycles of 5 min hypoxia (12%) followed by 5 min normoxia, 3 sessions/week for 3 weeks	Reduced blood glucose (fasting and OGTT), associated with HIF-1α
(Fuller and Courtney, 2016)	A female patient (age 49) with obesity and pre-diabetes	IH: daily hour-long session of alternating 6 min hypoxia and 3 min normoxia	Weight loss and glycemic control
(Morishima et al., 2015)	21 sedentary men (age 24.3 ± 1.2)	Hypoxic training (15%) for 2 weeks or 4 weeks	Improving insulin sensitivity
(Duennwald et al., 2013)	14 patients with type 2 diabetes	1 h single bout IH: 5 min hypoxia (13%) followed by 6 min normoxia	Reduced blood glucose

DBP, diastolic blood pressure; HTN, hypertension; HIF-1α, hypoxia-inducible factor-1alpha; HDLc, high-density lipoprotein cholesterol; IH, intermittent hypoxia; IHH, intermittent hypoxia-hyperoxia; LDLc, low-density lipoprotein cholesterol; NO, nitric oxide; NOx, nitric oxide metabolites; OGTT, oral glucose tolerance test; PDK-1, pyruvate dehydrogenase kinase 1; SBP, systolic blood pressure.

**TABLE 2:** Effects of intermittent hypoxia on vascular risk factors in animals.

References	Subjects	Hypoxia protocols	Results	Mechanisms
(Chen et al., 2021)	SHR and WKY rats	Hypobaric hypoxia (4000 m altitude), 5 h/day for 35 days	ABP↓	Inhibiting RAS activity, downregulating the ACE-Ang II-AT1 axis, upregulating the ACE2-(Ang17)-Mas axis
(Li et al., 2019)	RVH rats	Hypobaric hypoxia (5000 m altitude), 6 h/day for 28 days	ABP↓	Upregulating NOS expression in the nucleus tractus solitarii
(Manukhina et al., 2011)	SHR rats	5–10 min hypoxia (9.5–10%) and 4 min normoxia, 5–8 cycles/day for 20 days	Suppressed the development of hypertension	Prevention of endothelial dysfunction, increased accumulation of NO stores in vascular walls
(Luo et al., 2022)	C57BL/6L mice with HFD	10% oxygen for 1h/day, 4 weeks	Reduce body weight; ameliorate fatty liver	Associated with hypoxia-induced epinephrine
(Tian et al., 2016)	Sprague–Dawley rats	Hypobaric hypoxia (simulate 5000 altitudes) for 6 h/day, 4 weeks	Decreased SAP, serum triglyceride and cholesterol; improved insulin resistance and hepatic steatosis	Ameliorating insulin resistance *via* the HIF-insulin signaling pathway
(Trzepizur et al., 2015)	C57BL/6J mice with HFD	IH: 1 min cycle, hypoxia (5% O_2_, 30 s) followed by normoxia (21% O_2_, 30 s) for 8 h/day, 2 weeks	Increase insulin and leptin levels; restore endothelial function and mitochondrial activity; limits liver lipid accumulation	Prevented endothelial dysfunction by restoring NO production; improved liver lipid metabolism by restoring mitochondrial activity

ABP, arterial blood pressure; ACE, angiotensin-converting enzyme; Ang, angiotensin; HFD: high-fat diet; HIF, hypoxia-inducible factor; IH, intermittent hypoxia; NO, nitric oxide; NOS, nitric oxide synthase; RAS, renin-angiotensin system; SHR, spontaneously hypertensive rat; WKY, Wistar-Kyoto.

### Intermittent hypoxia and abnormal glucose and lipid metabolism and obesity

Modern lifestyle frequently entails lack of exercise and high-calorie dietary intake, leading to an increasing number of cases of type 2 diabetes and obesity (Ampofo and Boateng, 2020; Sun et al., 2022), which are, in turn, associated with increased morbidity and mortality. Clinical evidence shows that IH can improve abnormal glucose and lipid metabolism in humans (Table 1). A single session of IH can improve cardiopulmonary reflexes and exert a hypoglycemic effect in patients with type 2 diabetes (Duennwald et al., 2013). Moderate IHT lasting for 3 weeks is potentially useful for the management of patients with pre-diabetes and can induce an increase in the expression of HIF-1α mRNA and its target gene, which can be used as an effective non-pharmacological preventive therapy (Lyamina et al., 2011). Another study confirmed that IHT can restore blood insulin levels to normal levels in prediabetic patients, and this was related to the increased expression of PDK-1 mRNA in leukocytes (Serebrovska et al., 2019b). Additionally, in one case, a female patient with obesity and pre-diabetes not only experienced controlled blood glucose but also weight loss through IH treatment (Fuller and Courtney, 2016). For sedentary individuals, 4 weeks of IHT improved insulin sensitivity more than 2 weeks of the same number of IHT sessions, suggesting that a longer IHT schedule may be more beneficial for improving insulin sensitivity (Susta et al., 2020). IHT also lowered blood pressure in this study, but this was not related to the duration of training. In overweight or obese adults, 2 weeks of passive moderate IH improved cardiovascular risk factors by lowering blood glucose, low-density lipoprotein, and cholesterol (Costalat et al., 2018). Three weeks of IHH treatment for patients with metabolic syndrome can improve the blood lipid profile and anti-inflammatory state (Afina et al., 2021). IHH therapy is safe and well-tolerated by patients, can reduce arteriosclerosis, and positively affect liver function by improving the hemodynamics and lipid profile of patients (Bestavashvili et al., 2022). The specific mechanism by which hypoxia improves glucose and lipid metabolism remains unclear. Animal studies have shown that the regulatory mechanism of IH on metabolism may be related to hypoxia-induced epinephrine (Luo et al., 2022), ameliorating insulin resistance *via* the HIF-insulin signaling pathway (Tian et al., 2016), and recovery of mitochondrial activity (Trzepizur et al., 2015; Table 2).

Taken together, these results suggest that IH can improve blood pressure, blood glucose, blood lipids, and weight loss; providing a new therapeutic strategy for the treatment and prevention of atherosclerosis and metabolic syndrome. However, more evidence from randomized controlled trials and animal experiments is needed to support these conclusions. Moreover, the above-mentioned clinical and preclinical studies (Tables 1, 2) suggest that IH can improve conditions that are common risk factors for IS, which is not only beneficial to the prevention of cerebrovascular diseases but also helpful to reduce the likelihood of recurrence.

## Application of intermittent hypoxia conditioning at different stages of ischemic stroke

**FIGURE 2:**
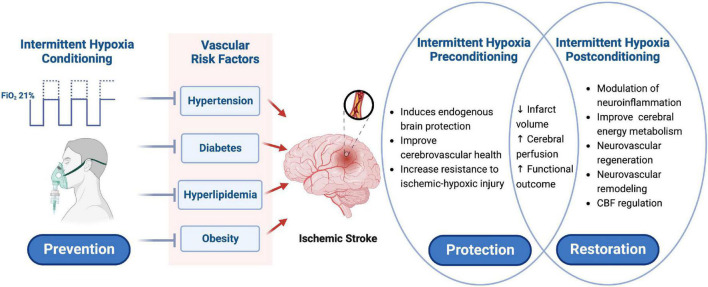
Schematic presentation of intermittent hypoxia conditioning as a potential prevention and treatment strategy for ischemic stroke. IH conditioning can improve the conditions considered as risk factors for cerebrovascular diseases, such as high blood pressure, glucose, and lipid content, and high body weight, which is a potential treatment strategy to prevent IS and reduce its incidence and recurrence rate. IH preconditioning induces endogenous cerebral protective mechanisms, improves cerebrovascular health, and enhances the resistance of the brain to ischemic-hypoxic injury. IH postconditioning can modulate neuroinflammation, improve brain energy metabolism, promote neurovascular regeneration and remodeling, and regulate cerebral blood flow. Applying IH conditioning at different stages of IS may ultimately reduce infarct volume, improve cerebral perfusion, and promote neurological recovery. IH, intermittent hypoxia; IS, ischemic stroke. Created with www.biorender.com.

The potential therapeutic use of IH in the treatment of cerebrovascular and cardiovascular diseases is the focus of extensive research (Manukhina et al., 2016; Serebrovskaya and Xi, 2016; Mallet et al., 2018). The mechanisms underlying the beneficial effects of hypoxia adaptation have been investigated at multiple biological levels, ranging from systemic physiological responses to genomic regulation and protein modifications (Terraneo et al., 2017; Lee et al., 2020; Burtscher et al., 2022). Preconditioning is a therapeutic strategy that induces endogenous self-protection of vital organs through sub-lethal physiological stimulation (Stevens et al., 2014). Experimental studies in rodents have shown that IH can trigger beneficial effects through preconditioning, improving the brain and heart’s defenses against ischemic-hypoxic injury and protecting them from the harmful consequences of ischemia and reperfusion (Stowe et al., 2011; Manukhina et al., 2013). Postconditioning refers to the promotion of recovery from injury by promoting processes such as repair, regeneration, and plasticity, which contribute to an improved prognosis (Pietrogrande et al., 2019; Li et al., 2022). Therefore, the treatment approach of IH preconditioning and postconditioning at different stages of the disease can not only reduce risk of stroke but also enhance neuroprotection to reduce the injury severity and ultimately improve the prognosis of IS (Figure 2).

### Intermittent hypoxia preconditioning

Experimental studies in rodents show that hypoxia preconditioning before an acute IS can induce endogenous brain protection, increase the brain’s resistance to ischemic-hypoxic injury, and produce a neuroprotective effect by activating the hypoxic signal pathway, the anti-inflammatory pathway, antioxidative stress, and autophagy (Liu et al., 2021). How to maximize the protective effect and obtain the greatest benefit depends on the mode of hypoxia. Animal studies have found that either a single episode of IH (Miller et al., 2001) or consecutive days of IH (Lin et al., 2003) reduce the infarct size in a transient focal IS model and have a neuroprotective effect. However, this protective effect diminishes over time. Repeated IH with different concentrations and durations can provide sustained brain protection by regulating epigenetic neurovascular plasticity, inducing a longer tolerance time window (Stowe et al., 2011; Monson et al., 2014; Selvaraj et al., 2017). It is worth noting that IH preconditioning has a protective effect against IS, and hypoxic concentration is the key factor. Studies have confirmed that 10% of IH is neuroprotective, whereas 6% of IH exacerbates tissue damage, and this different outcome is associated with changes in susceptibility to mitochondrial damage (Jackman et al., 2014). A recent study confirmed for the first time that brief, repeated exposure to systemic hypoxia attenuates hypoperfusion-induced cognitive impairment and that this resistance to dementia is heritable, allowing mice offspring to avoid memory loss even in the presence of persistent cerebral hypoperfusion (Belmonte et al., 2022). Due to the unpredictability of IS, few clinical studies have focused on it, with most studies conducted on animal models. Therefore, future research needs to strengthen our understanding of the neuroprotective mechanism of preconditioning in the brain and find the optimal hypoxia mode and applicable population to accelerate clinical translation.

### Intermittent hypoxia postconditioning

Hypoxia postconditioning, which is administered during the acute phase of IS, can have a protective effect by improving cerebral energy metabolism (Ren et al., 2020). It also stimulates neural regeneration after IS by promoting the proliferation and migration of neural stem cells (Li et al., 2022). Hypoxia postconditioning 48 h after an IS can improve motor function and reduce tissue loss (Pietrogrande et al., 2019). In addition, the adaptation after hypobaric hypoxia in the acute stage can also improve neurological function and reduce the infarct volume after IS, which is related to the fact that hypobaric hypoxia attenuates the inflammatory response by regulating the expression of HIF-α and its target genes (Huang et al., 2014; Qiao et al., 2015). In contrast, some studies have shown that hypoxic postconditioning in the acute phase of IS does not improve neurological function but may increase brain damage instead (Tsai et al., 2008). These differences in results may be related to the dose of hypoxia and the animal model used in each case. A subsequent study, found that hypoxia postconditioning in the chronic stage of IS can improve cognitive dysfunction in mice after stroke by inducing hippocampal neurogenesis and functional synapse formation (Tsai et al., 2013), indicating that hypoxia postconditioning can promote brain repair after injury.

To summarize, based on the current evidence from experimental and clinical studies, IH preconditioning may not only prevent cerebrovascular diseases and reduce their recurrence by controlling risk factors (Tables 1, 2) but also reduce post-stroke brain injury and neurological deficit by activating the adaptive response of the brain (Table 3). IH postconditioning may promote neurological recovery (Table 4) and the regulation of cerebral blood flow through neurovascular remodeling (Manukhina et al., 2016). Moreover, IH may improve the conditions commonly identified as risk factors for stroke, promote repair and regeneration after injury, and play a role in preventing the recurrence of stroke and reducing the brain tissue injury after recurrence, thus reducing the disability and mortality rates of the disease, and its burden as well. Therefore, IH can be used in the prevention, treatment, and rehabilitation phases of IS and is a very promising adjunctive therapy. However, the present evidence supporting its use for the treatment during the acute recovery phases is limited to a few animal studies and is, therefore, not yet sufficient. Future studies need to further elucidate the neuroprotective and neurorestorative mechanisms of hypoxia conditioning while also considering the effects of age, sex, comorbidities, and the safety and efficacy of its application during the acute and recovery phase, to help accelerate its clinical translation.

**TABLE 3:** Beneficial effects of intermittent hypoxia preconditioning in ischemic stroke.

References	Subjects	Model	Hypoxia protocols	Outcomes
(Belmonte et al., 2022)	C57BL/6J mice	Chronic cerebral hypoperfusion	Hypoxia (11%) 1 h, every other day for 8 weeks	Abrogated hypoperfusion-induced memory/plasticity deficits, resistance to dementia is heritable
(Jackman et al., 2014)	C57BL/6J mice	tMCAO	Hypoxia (10 or 6%) 90 s followed by normoxia 90 s, 20 hypoxic episodes per hour	10% CIH is neuroprotective (reduced infarct volume), 6% CIH exacerbates tissue damage
(Stowe et al., 2011)	Swiss-Webster ND4 mice	tMCAO, pMCAO	2 weeks of repetitive hypoxic (8–11%) preconditioning (RHP) or single hypoxic preconditioning (SHP; 4 h, 8% O_2_)	RHP protection against stroke persisted for 8 weeks
(Lin et al., 2003)	Wistar rats	tMCAO	Hypoxia (380 mmHg altitude) for 15 h/day, lasted 4 weeks	Reduced infarct volume
(Miller et al., 2001)	C57Bl/6, 129SvEv, Swiss-Webster ND4 mice	tMCAO	48 h before tMCAO, hypoxia (11%) for 2 h	Reduced infarct volume

CIH, chronic intermittent hypoxia; pMCAO, permanent middle cerebral artery occlusion; RHP, repetitive hypoxic preconditioning; tMCAO, transient middle cerebral artery occlusion.

**TABLE 4:** Beneficial effects of intermittent hypoxia postconditioning in ischemic stroke.

References	Subjects	Model	Hypoxia protocols	Outcomes
(Su et al., 2022)	SD rats	tMCAO	1 week after stroke, IH (13% O_2_) for 4 h/day, lasted 4 weeks	Reduce infarct volume and promote motor function recovery
(Li et al., 2022)	C57BL/6 mice	tMCAO	30 min after stroke, IH (8% O_2_) for 3 h/day, lasted 13 days	Recovery of neurological function, promote the proliferation and migration of neural stem cells
(Ren et al., 2020)	C57BL/6J mice	dMCAO	15 min after stroke, IH repeated four times	Reduce infarct size
(Pietrogrande et al., 2019)	C57BL/6 mice	Photothrombotic occlusion	48 h after stroke, IH (11% O_2_) for 8 h/day, lasted 14 days	Reduce motor deficits and tissue loss
(Qiao et al., 2015)	C57 mice	MCAO	24 h after stroke, IH (simulate 5000 m altitude) for 4 h, lasted 7 days	Accelerate cognitive function recovery
(Huang et al., 2014)	C57BL/6 mice	tMCAO	12 h after stroke, IH (simulate 5000 m altitude) for 4 h	Better neurological performance and smaller infarct size
(Tsai et al., 2013)	SD rats	MCAO	7 days after stroke, IH (12% O_2_) for 4 h/day, lasted 7 days	Alleviate long-term memory impairment
(Leconte et al., 2009)	SWISS mice	tMCAO	5 days after stroke, IH (8% O_2_) for 1 h/day, 3 times/week, lasted 43 days	Reduced delayed thalamic atrophy

IH, intermittent hypoxia; pMCAO, permanent middle cerebral artery occlusion; tMCAO, transient middle cerebral artery occlusion.

## Therapeutic effects and mechanism of hypoxia conditioning on ischemic stroke

Ischemic stroke is the most common acute cerebrovascular disease, with complex pathophysiological mechanisms and high heterogeneity. Severe ischemia after vascular occlusion leads to rapid brain injury and cell death, which activates the immune system *in vivo* (Iadecola et al., 2020). Inflammatory signals participate in all stages of the ischemic cascade reaction, leading to early blood-brain barrier (BBB) destruction and infarction progress, but also to later neurovascular repair and remodeling. Like the complex pathophysiological mechanisms of cerebral ischemia, the protective mechanisms underlying hypoxia conditioning are diverse and intertwined, and have not been fully elucidated. The concept that hypoxia can induce an inflammatory response has been widely recognized from the study of the hypoxia pathway (Eltzschig and Carmeliet, 2011), suggesting that beneficial hypoxic modulation can modulate the body’s immunity, not only by reducing early inflammatory damage but also by promoting the repair process after brain injury through anti-inflammatory effects. In addition, it has been shown that hypoxia is involved in neurovascular repair (Walshe and D’Amore, 2008), generation of cerebral collateral vessels (Anan et al., 2009; Zhang et al., 2020), and capillary neovascularization (Milner et al., 2008; Burnier et al., 2016), neurogenesis, and neuroplasticity (Sommer, 2009; Zhu et al., 2010; Tsai et al., 2013; Li et al., 2022) after IS. Therefore, moderate hypoxia conditioning may improve the clinical outcome of IS by regulating these processes to promote endogenous brain repair and regeneration.

### Hypoxia and neurovascular protection

The neurovascular unit (NVU) is the structural and functional unit of the central nervous system. It highlights the dynamic interactions between endothelial cells, mural cells (pericytes and smooth muscle cells), basement membrane, astrocytes, microglia, neurons, and extracellular matrix; and the importance of such interactions in the pathophysiology of the CNS (Schaeffer and Iadecola, 2021). The capillary network of the whole brain is composed of endothelial cells and connected by tight junctions, surrounded by the endfeet of pericytes and astrocytes, constituting an important part of the BBB and maintaining brain homeostasis (Liebner et al., 2018). The close communication between microvascular endothelial cells and surrounding astrocytes plays an important role in maintaining intact neurovascular coupling (Iadecola, 2017). Moreover, the regulation of the microvascular basement matrix membrane, the activation of endothelial cells, and the change of glial cell adhesion directly affect the transmission of information between microvessels and neurons. The organism relies on this complex neurovascular network to achieve the fine regulation of cerebral blood flow and ensure the normal neurological function and steady state of the brain (Zhang et al., 2012). It has been confirmed that IH preconditioning can significantly reduce oxidative stress during ischemia-reperfusion injury and stimulate NO-induced vasodilation (Bertuglia, 2008), thereby maintaining capillary perfusion. In the large vessels, periodic IH increases the blood flow and shear rate of the internal carotid artery, which in turn increases shear-mediated vasodilatation (Iwamoto et al., 2020). It is suggested that hypoxia can improve cerebral blood flow and cerebral perfusion by regulating the diameter of cerebral vessels. In addition, the oxygen uptake capacity of the brain increases during acute periodic hypoxemia to compensate for the reduced oxygen levels in arterial blood (Liu et al., 2017). Repeated IH exposures enhance arterial oxygen delivery and increase O_2_ availability (Zhang et al., 2010). These results suggest that hypoxia conditioning can not only affect cerebral vascular reactivity, but also increase cerebral oxygen uptake, oxygen transport, and oxygen use capacity. The studies mentioned above suggest that periodic IH is a promising non-pharmacological treatment strategy for optimizing cerebrovascular health.

Under physiological conditions, hypoxia conditioning can regulate the interactions between various cellular and non-cellular components in the NVU through HIF-dependent or independent pathways (Obrenovitch, 2008; Terraneo et al., 2017; Lee et al., 2020), modulate the organism’s response to injury, enhance the resistance of the NVU to ischemic-hypoxic injury, and exert neuroprotective effects (Dirnagl et al., 2003; Stowe et al., 2011, 2012; Wacker et al., 2012a,b; Monson et al., 2014; Selvaraj et al., 2017). HIFs are the major regulators of the hypoxic transcriptional response, and the O_2_-sensitive prolyl/aspartate hydroxylase (PHDs/FIH) regulates HIF activity in the transition between normoxia and hypoxia conditions (Kaelin and Ratcliffe, 2008). Under various pathological conditions, hypoxia conditioning serves to improve the clinical outcome of the disease by regulating the cellular environment (Taylor and Colgan, 2017), thereby enhancing mitochondrial metabolism, enhancing antioxidant and anti-inflammatory capacity, and promoting the repair process to reduce the extent of neurological and vascular damage (Walshe and D’Amore, 2008; Tsai et al., 2013; Qiao et al., 2015; Ryou et al., 2017; Manukhina et al., 2018; Pietrogrande et al., 2019; Ren et al., 2020; Li et al., 2022; Luo et al., 2022). Since the underlying cellular and molecular mechanisms are unclear, more relevant research evidence is needed.

### Hypoxia and neurovascular regeneration

Cerebral collateral circulation refers to the auxiliary vascular network recruited after arterial occlusion, which can provide partial blood flow compensation for the ischemic area (Liebeskind, 2003). Cerebral collateral vessels refer to the inherent vascular anastomosis among arteries, arterioles, and capillaries (Faber et al., 2014). When cerebral artery stenosis or occlusion leads to ischemia of its downstream brain tissue, good collateral vessel opening can play a role in blood flow compensation. In IS, collateral blood perfusion in the area adjacent to occluded vessels can partially alleviate the ischemic injury caused by insufficient perfusion (Ginsberg, 2018). However, due to the variation in number, diameter, and compensatory capacity of collateral vessels, the degree of protection of collateral circulation against occlusive diseases varies greatly, which directly affects the clinical outcome of patients. Factors affecting collateral vessels include aging (Faber et al., 2011; Ma et al., 2020), genetic background (Lucitti et al., 2016; Faber et al., 2021), and vascular risk factors that can be treated (Biose et al., 2020), including hypertension (Moore et al., 2015), type 2 diabetes (Akamatsu et al., 2015; Nishijima et al., 2016), dyslipidemia, obesity, and metabolic syndrome (Menon et al., 2013). The above-mentioned factors can cause the thinning of collateral vessels and the impairment of the compensation ability of collateral blood flow. The underlying mechanism remains unclear, but may be related to the decrease in eNOS levels and an increase in oxidative stress and inflammation (Faber et al., 2011; Moore et al., 2015; Rzechorzek et al., 2017). Hypoxia conditioning may improve the status of the collateral circulation by improving NO utilization by endothelial cells, antioxidative stress mechanisms, and anti-inflammatory effects (Anan et al., 2009). In addition, mild hypoxia can promote endothelial cell proliferation, increase vascular density, and remodel capillaries and arterioles in mice (Milner et al., 2008; Boroujerdi et al., 2012; Burnier et al., 2016; Halder et al., 2018). Recently, the formation of new collateral vessels induced by arterial occlusion has been confirmed in mouse models for IS and myocardial infarction (Zhang and Faber, 2015; Okyere et al., 2020). Furthermore, it has also been reported that exposure to hypoxia alone can induce the formation of new collateral vessels in the brain and the heart (Zhang et al., 2020; Aghajanian et al., 2021). By gradually acclimating mice to hypoxia and maintaining it for 2–8 weeks, oxygen concentration-dependent new collateral vessel formation and remodeling of intrinsic collateral vessels were observed, and cerebral infarct volume was reduced after subsequent permanent middle cerebral artery occlusion. The expression of Hif2α, Vegfa, Rabep2, Angpt2, Tie2, and Cxcr4 increased after hypoxia. However, in knockout mice for Rabep2, new collateral vessels could not be formed, and this phenomenon was reversed in the conditional knockouts for Vegfa, Flk1, and Cxcr4 (Zhang et al., 2020). Recently, it has also been found that hypoxia can induce the formation of coronary collaterals in adult mice, and that Vegfa and Rabep2 are required for this (Aghajanian et al., 2021). These results suggest a mechanistic link between embryonic collateral formation and new collateral formation in adult mice. How collateral vessels are formed and how hypoxia promotes collateral vessel neovascularization and remodeling are questions that need to be addressed in the future.

Adult neurogenesis is of great medical significance to cognitive, memory, and motor dysfunction caused by central nervous system diseases (Culig et al., 2022). Experimental evidence shows that intermittent hypobaric hypoxia can promote the proliferation of endogenous neural progenitor cells, leading to an increase in the number of new neurons, and produces antidepressant-like effects. IH can also promote the expression of brain-derived neutrophic factor in the adult hippocampus (Zhu et al., 2010). In a rat model for Alzheimer’s disease, IH conditioning protected against neurodegenerative changes and improved cognitive function (Manukhina et al., 2010). The benefits of IH for improving cognitive function have been further confirmed in human studies. Schega et al. (2013) reported for the first time that additional IH conditioning administered before physical exercise can enhance cognitive function and quality of life in the older adults, and demonstrated good tolerance. Wang et al. (2020) reported that moderate IH for eight weeks can reduce arterial blood pressure at rest, enhance cerebral oxygenation and vasodilation in the cerebral cortex during hypoxia, and improve the short-term memory and attention of older adults patients with amnestic mild cognitive impairment. Serebrovska et al. (2019c) showed that IHH conditioning can improve the cognitive function of patients with mild cognitive impairment and reduces the biomarkers for Alzheimer’s disease in the peripheral blood while increasing the levels of some inflammatory markers. The upregulation of inflammatory markers may be a potential trigger for cellular adaptation (Serebrovska et al., 2022), but whether these proinflammatory factors mediate the neuroprotective effects is unclear and needs to be further explored.

However, due to the unpredictability of this acute cardio-cerebrovascular disease, the clinical research evidence on hypoxia preconditioning is insufficient. Most rodent studies have been conducted in young healthy mice; but in the clinical setting IS patients are usually middle-aged or older adults, often affected by comorbid hypertension, diabetes, dyslipidemia, and obesity; which complicates the pathology of IS (Candelario-Jalil and Paul, 2021). Whether the same pattern of hypoxia preconditioning can also provide the an equivalent protective effect to aging individuals exhibiting comorbid conditions lacks support from relevant research evidence and needs to be further clarified in future studies. More systematic and comprehensive studies are needed in the future to enhance our understanding of the potential molecular and cellular regulatory mechanisms underlying the beneficial effects of hypoxia, and to provide a strong basis for the application of hypoxia conditioning as a clinical treatment for IS at different stages.

## Bridging the gap between preclinical and clinical studies in ischemic stroke: A translational perspective

The current research results provide promising evidence for the application of IH conditioning in the prevention, treatment, and rehabilitation of IS. It should be noted that the inconsistency between the results obtained in rodent models and in the clinical population may affect the translational outcome of IH treatment, and these factors include genetics, age, sex, comorbidity, etc. (Sommer, 2017; Candelario-Jalil and Paul, 2021). In recent years, researchers have noticed that the opposite circadian rhythm between rodents and humans also affects the outcome of stroke treatment (Esposito et al., 2020; Lo et al., 2021). Therefore, it is necessary to optimize the preclinical models of IS to simulate as closely as possible what happens in the actual clinical patients. Although many clinical and experimental studies associated with normobaric IH have been reported, the protocol of hypoxia treatment varies considerably between individual studies. It is not clear which mode of treatment is the best. Therefore, searching for potential clinical biomarkers that can identify hypoxia adaptation and maladaptation may help to provide more accurate and safer IH treatment.

### The effect of circadian rhythm

A growing body of research has shown that circadian rhythms interact with multiple aspects of IS pathophysiology, influencing disease susceptibility, degree of damage, repair processes, and response to various treatments, but the underlying mechanism is still unclear (Lo et al., 2021). Recent evidence suggests crosstalk between HIF-1 signaling and the circadian rhythm (Manella et al., 2020; Adamovich et al., 2022). Esposito et al. (2020) reported that three different neuroprotective methods can reduce infarction in rodent models during the day, but not at night, pointing out that circadian rhythm may be one of the factors affecting the clinical translation of neuroprotection. This study suggests that the response of the body to treatment is different in the active and in the inactive phases, and that the diurnal effect may lead to different outcomes. Moreover, it has also been found in clinical studies that aspirin, a drug used to treat IS, can be orally administered before sleep to reduce platelet reactivity upon waking, which is related to the endogenous circadian rhythm of platelet activation (Bonten et al., 2015). A recent study of circadian gene expression profiles in 12 different mouse organs showed that circadian rhythms exist in the transcription of 43% of protein-coding genes and that most of the best-selling and WHO essential drugs directly target genes that are regulated by circadian rhythms (Zhang et al., 2014). Therefore, it can be speculated that different administration times will have different effects on treatment outcomes and may be beneficial to the treatment of various diseases. Therefore, the effect of the timing of the intervention on the treatment outcome should be considered in preclinical studies of hypoxia conditioning. To our knowledge, no studies on experimental animals or humans have been published in this area, and circadian factors could be included in subsequent studies to provide a basis for the clinical translation of hypoxia conditioning.

### Optimizing the preclinical model of ischemic stroke

It is well-known that many differences between clinical and experimental study subjects have resulted in potential treatments identified as promising in rodent IS models that have not shown therapeutic effects in clinical trials. Clinically, stroke is most prevalent in older men and women, and preclinical studies have mostly tested young male animals. Therefore, preclinical studies in animal models of IS affected by aging and comorbidities can more accurately simulate the clinical situation of patients. Aging is a natural phenomenon in which mitochondrial dysfunction, oxidative stress, and inflammation gradually increase with age, resulting in different cellular dysfunctions and a progressive decline in tissue and organ function, which leads to an increased risk of IS and death (Garaschuk et al., 2018). Preclinical studies have also revealed significant differences in the pathophysiology and prognosis of IS between young and old animals, with the latter exhibiting more severe injury and poor recovery. The cellular mechanisms of aging and IS overlap, and beneficial hypoxia conditioning can inhibit mitochondrial dysfunction, oxidative stress, and inflammation, and potentially provide effective treatment for IS among older adults (Burtscher et al., 2021a). In experimental studies, female rodents were found to have milder damage after IS compared to males. This phenomenon disappeared after ovariectomy or beyond reproductive age, suggesting that estrogen and progesterone are potential neuroprotective factors against IS (Alkayed et al., 2000). Uric acid failed to act as a neuroprotective agent in patients with acute IS, but it had a positive effect on functional recovery in female patients (Chamorro et al., 2014). Given the age and sex differences described above, it is necessary to clarify the effect that these factors may have on treatment outcomes in animal models before conducting clinical trials to further evaluate the efficacy and safety of IH treatment. Additionally, common comorbidities such as hypertension, diabetes, hyperlipidemia, obesity, etc. significantly increase the vulnerability of the brain to ischemic injury, eventually leading to worse functional results (Biose et al., 2020; Candelario-Jalil and Paul, 2021). Under the condition of comorbidity and aging, the protective effect of some drugs is weakened or absent. Therefore, whether IH can also induce beneficial effects among older adults affected by multiple comorbidities is still unclear and needs to be further explored.

## Conclusion and prospects

Intermittent hypoxia conditioning is a very promising treatment strategy to prevent and treat IS (Figure 2). IH can improve the conditions considered to be risk factors for cerebrovascular diseases, such as blood pressure, blood sugar, blood lipid, and weight, and increase the body’s resistance to ischemic-hypoxic injury. Therefore, IH may reduce the incidence and recurrence of IS and have a protective effect on the central nervous system, thereby reducing the severity of IS and improving the clinical prognosis of patients. According to the evidence detailed in this review, IH conditioning can reduce damage to the BBB and neuronal cells, and can also induce neovascularization and neurogenesis, contributing to NVU repair and regeneration. Thus, during the recovery phase of IS, IH conditioning promotes neurovascular remodeling, which is beneficial to transition the brain from injury to the repair process. Future research must further clarify the cerebral protective mechanisms of IH conditioning and other beneficial mechanisms, which will help to provide new therapeutic strategies and potential pharmacological targets for the prevention and treatment of cerebrovascular diseases. In addition, issues that need to be solved in future research include the optimal intervention mode, the timing of intervention, the difference in circadian rhythms, sex, age, and comorbidities in response to IH, and the different responses of different brain regions and cell types to IH. At present, IH conditioning for ischemic cerebrovascular disease has been almost exclusively studied in preclinical, animal disease models, and the challenge for the future is how to apply it safely and effectively for the prevention and treatment of IS in clinical patients. Therefore, well-designed controlled clinical trial studies are needed to confirm these findings and determine the optimal target population, time points for intervention, and mode of hypoxia; and ultimately to provide strong evidence for establishing the most effective individualized hypoxia protocol.

## Author contributions

HY investigated and wrote the manuscript. JL reviewed and contributed to the editing.YG participated in the proofreading. XJ and GN contributed to the conception and critically revised the manuscript. All authors have read and agreed to the published version of the manuscript.
